# *Trans*porting the Burden of Justification: The Unethicality of Transgender Conversion Practices

**DOI:** 10.1017/jme.2022.85

**Published:** 2022

**Authors:** Florence Ashley

**Affiliations:** 1. UNIVERSITY OF TORONTO FACULTY OF LAW AND JOINT CENTRE FOR BIOETHICS, TORONTO, CANADA

**Keywords:** Conversion Practices, Conversion Therapy, Gender Transition, Transgender, Clinical Ethics, Pathologization

## Abstract

Transgender conversion practices involve attempts to alter, discourage, or suppress a person’s gender identity and/or desired gender presentation, including by delaying or preventing gender transition. Proponents of the practices have argued that they should be allowed until proven to be harmful. Drawing on the notion of expressive equality, I argue that conversion practices are prima facie unethical because they do not fulfill a legitimate clinical purpose and conflict with the self-understanding of trans communities.

## Introduction

Transgender conversion practices aim to alter, discourage, or suppress a person’s gender identity and/or desired gender presentation, including by delaying or preventing gender transition.^1^ Conversion practices are heterogeneous and wide-ranging. They include not only wanting to help individuals “reconcile with their natal body,” but also attempts to identify the cause of the person’s expressed gender — including under the pretext of gender exploration — pursuant to the belief that it may be caused by social contagion, trauma, mental illness, internalized homophobia, and flight from womanhood.^2^ Tying together these heterogeneous practices is the belief that transitude — being trans — is suspect, and that cisgender identities are more desirable, legitimate, or authentic.^3^ Although some governments have banned conversion practices targeting gender identity, it remains legal in most jurisdictions. Instead of falling into desuetude, trans conversion practices may experience a revival in the wake of recent legislative and judicial attempts to curtail access to gender-affirming care.^4^

Despite widespread condemnation by professional organizations, some theorists and practitioners defend trans conversion practices on account of the fact that their harmfulness has not been proven, unlike that of gay conversion practices, and therefore argue that trans conversion practices should be allowed.^5^ Underlying this argument is the premise that opponents of trans conversion practices bear the ethical burden of justification and must prove the practices’ harmfulness before they can be deemed unethical and prohibited. In this paper, I argue that the argument misrepresents the ethical burden of justification. Trans conversion practices are *prima facie* unethical because they are contrary to the ideals of equality and justice. Accordingly, their proponents carry the burden of establishing that they are significantly more beneficial than all alternative clinical approaches — enough to outweigh that inequality. Since the available evidence does not countenance the view that trans conversion practices have better outcomes, we must conclude that they are unethical.

To be sure, we have reasons to believe that trans conversion practices are psychologically harmful. However, given the egalitarian implications of trans conversion practices, is sufficient to prohibit them that we have no evidence that they have substantially better outcomes than other approaches, such as gender-affirmative approaches. That the burden of justification lies on proponents of trans conversion practices is meaningful, because it allows us to circumvent methodological debates around whether the evidence of harm is strong enough to justify prohibition.

In this paper, I argue that the argument misrepresents the ethical burden of justification. Trans conversion practices are *prima facie* unethical because they are contrary to the ideals of equality and justice. Accordingly, their proponents carry the burden of establishing that they are significantly more beneficial than all alternative clinical approaches — enough to outweigh that inequality. Since the available evidence does not countenance the view that trans conversion practices have better outcomes, we must conclude that they are unethical.

The paper is divided into four sections. The first section draws on the work of legal philosopher Paul Gowder to establish expressive equality as an ethical requirement for clinical practices. The second section argues that conversion practices are *prima facie* unethical due to their negative relationship to expressive equality. The third section demonstrates that conversion practices do not overcome this *prima facie* case given the available evidence. The fourth section explores the implications of my arguments for the law and other clinical practices.

## Expressive Equality as an Ethical Requirement

In his book *The Rule of Law in the Real World*, legal philosopher Paul Gowder defends an expressivist vision of the rule of law.^6^ Unlike conceptions of the rule of law that require statutes and government policies to be general in a formal rather than substantive manner, Gowder argues that laws are only sufficiently general if they are justified by reasons that we can reasonably expect everyone to accept. His position is reminiscent of John Rawls’ doctrine of public reason, according to which individuals exercising political power must act according to reasons that free and equal individuals might reasonably endorse.^7^ It also resonates with Joseph Raz’s conception of authority, which is justified when those subject to the authority are more likely to act in accordance with reasons that apply to them by following the authority’s lead than by following their own judgment.^8^ Establishing whether reasons are justified requires us to inquire into the expressive content of the law, as laws will only be general in the relevant sense if the message communicated by the law is “consistent with conceiving of all members of the community as free and equal,” which I call expressive equality.^9^

The expressive content of a law or policy emerges from considering three points of view. From the first-person standpoint, what attitudes must policymakers hold to rationally enact the law or policy for some public purpose? From the second-person standpoint, what attitudes must those impacted by the policy hold in order to understand the law as helping them act according to reasons that already apply to them? And from the third-person standpoint, what attitudes must the public hold in order to understand the law as enacted in their name and as expressing their self-understanding as a political community? The questions are answered through rational argument rather than by survey. However, the expressive content of policies supervenes on the sociocultural reality of relevant communities — making their perspectives informative even if not determinative. If a policy is inconsistent with conceiving of all members of the community as free and equal on any of the three standpoints, it violates expressive equality.

Although crafted with legislators and policymakers in mind, this framework can also be applied to professional conduct. Healthcare professionals, like legislators and policymakers, are afforded social and legal authority. This authority, bestowed by law, grants them significant influence over other individuals and society more generally in the hopes that they will act for the betterment of the public rather than under the yoke of idiosyncratic reasons. Their authority and orientation towards the public good coalesce into fiduciary duties that set them apart from ordinary social actors. Accordingly, they are subject to shared standards of behavior set by licensing and disciplinary bodies. Professional self-regulation “is a privilege and […] each [clinician] has a continuing responsibility to merit this privilege.”^10^

While clinicians’ foremost duty is to the patient, many of their obligations exceed the scope of the doctor-patient relationship. Professional codes of ethics recognize duties towards the profession, other professionals, and the public. For that reason, mental health professionals have a duty to report their patients in various situations involving risk to others, in violation of confidentiality and regardless of whether reporting is in the best interests of the patient.^11^

Mental health professionals are subject to norms of rationality and justice. Rationality lies at the heart of the scientific approach whereas commitment to justice is enshrined in leading documents of biomedical ethics. Justice is one of the four traditional principles of biomedical ethics, as set out by Tom Beauchamp and James Childress.^12^ It is also recognized as a foundational principle in the American Psychological Association’s *Ethical Principles of Psychologists and Code of Conduct*.^13^ For its part, the American Medical Association specifies that clinicians must avoid being influenced by “inappropriate considerations about […] gender identity.”^14^

Two objections may be raised to my argument that expressive equality should govern clinical practice. It may first be claimed that my analogy breaks down insofar as clinical relationships are freely chosen and contractual in nature, standing in stark contrast with the government’s ability to impose its views on others through coercion and force. According to this objection, the degree of authority borne by clinicians and policymakers so diverges in intensity that no analogy can be made between them. However, this objection overstates the differences between the two contexts. Involvement with governmental institutions is often voluntary, such as the decision of whether to marry or incorporate a business. At the same time, the voluntariness of clinical relationships is often severely undermined by the asymmetry of knowledge and power between clinician and patient, as recognized by the prohibition on sexual relationships between clinicians and patients. Clinicians may involuntarily commit individuals and are often trusted by patients because of their status. Such asymmetries are particularly stark in the context of trans conversion practices, where clinicians have been known to misrepresent the available evidence and professional consensus, disguise their practices in euphemistic language, and rely on the prejudices or confusion of parents or patients to secure ostensible consent. Trans individuals are particularly vulnerable to negative messaging due to internalized transantagonism, especially early in their journey.^15^

The second objection holds clinical approaches dissimilar to policymaking insofar as they are guided by principles of individualized care whereas government policies strive to be general. Good policies are general; good clinical care is individual. Thus, holds the objection, applying expressive equality — which is a part of the requirement of generality — to clinical care would be incoherent. This objection conflates generality with homogeneity. Governmental policies must be general inasmuch as they must be drafted for the whole of society, appealing to public reasons. A rule cannot single out named individuals for benefit or disadvantage — no rule could legitimately proclaim that Florence is the best, alas. Yet this does not preclude policies from taking into account differences within the population and allocating advantages accordingly. On the contrary, good policymaking *must* account for differences, for to treat all in the same manner without regard for the differences in their needs is to treat them unjustly. Much like clinicians, judges and administrative decision-makers are often afforded wide discretion in weighting the diverse factors and interests at play in any given situation to craft a response that is well adapted to the reality of the parties. Yet their decisions do not cease to be general in the sense of operating under reasons that we can reasonably expect everyone to accept. Generality and individualization are mutually reinforcing. If anything, professional licensing is one of the ways in which legislatures ensure individualized care through general schemes.

## Conversion Practices are *Prima Facie* Unacceptable

Expressive equality can be turned into a two-step analysis. First, we must ask whether conversion practices are *prima facie* inconsistent with conceiving of all members of the community as free and equal. The first-person and second-person viewpoints are most informative in relation to conversion practices, highlighting the perspective of clinicians and trans communities. Practices will be inconsistent with expressive inequality from the first-person viewpoint if it does not fulfill a legitimate clinical purpose recognized by the profession. Practices will be inconsistent with expressive inequality from the second-person viewpoint if it does not accord with trans communities’ self-understanding.

If conversion practices are inconsistent with expressive equality on either of the viewpoints, we then ask whether countervailing public reasons outweigh that *prima facie* inequality such that the practices would nevertheless be consistent with equality. For instance, conversion practices could theoretically betray prejudice towards transitude at first glance but ultimately be acceptable on account of being far more beneficial to patients than alternatives such as gender-affirming care. In this section, I argue that trans conversion practices are *prima facie* inegalitarian from both the first-person and second-person perspectives. In the next section, I show that they fail to outweigh this *prima facie* case based on the available evidence.

Trans conversion practices are not *prima facie* consistent with seeing transgender people as free and equal. Tying the disparate practices together is an assumption that trans lives are less authentic or desirable, constructing them as disordered and seeking to prevent them.^16^ The practices conflict with the American Psychological Association’s recognition that “diversity in gender identity and expression is part of the human experience and transgender and gender nonbinary identities and expressions are healthy, [and] incongruence between one’s sex [assigned at birth] and gender is neither pathological nor a mental health disorder.”^17^ The view of transitude as a positive form of human diversity, now adopted by professional organizations, has first emerged from trans communities and reflects their self-understanding. Attempts to unearth the reasons why a person might falsely believe themselves to be trans or wish to transition also betray a prejudicial view of transitude as suspect, in stark contrast to the presumption that cisgender identities are healthy and authentic. The pathologizing, negative, and distrustful impulses of conversion practices *prima facie* run afoul of expressive equality on both the first-person and second-person viewpoints.

### Legitimate Clinical Purpose

From the first-person viewpoint, trans conversion practices do not fulfill a legitimate clinical purpose recognized by the profession. While not determinative, professional consensus is strongly indicative of whether a practice serves a legitimate clinical purpose under the requirement of expressive equality. The quintessential clinical purpose is to cure, treat, or prevent a disorder. Historically, transitude was conceptualized as a psychopathology by psychiatry and psychology. Transitude was considered a mental disorder arising from disruptions in normal development and attributed to a variety of psychoanalytic causes such as separation anxiety, castration anxiety, oedipal complexes, lack of appropriately gendered role models, transference neurosis, schizophrenia, hysterical psychosis, obsessional interests, and escapist fantasizing.^18^ Mothers were also frequently blamed as sources of the child’s transitude for being gender non-conforming, unavailable, smothering, mentally ill, or having unresolved conflict and trauma.^19^ Clinicians rejected the suggestion that being trans may not be a disorder, arguing that the “required physical interventions are simply too radical to be thought about otherwise.”^20^ While some clinicians disallowed any and all patients from transitioning, others sought to distinguish those whose gender identity was irreversible and would benefit from transitioning from those who could still be “helped” and avoid transitioning. From 1980 to 2012, transitude was included in the *Diagnostic and Statistical Manual of Mental Disorders* under the labels of transsexualism and gender identity disorder.^21^

Clinical consensus has shifted since that era, however, and transitude is no longer understood as a disorder to be cured, treated, or prevented.^22^ In 2013, the fifth edition of the *Diagnostic and Statistical Manual of Mental Disorders* (DSM-5) replaced the category of gender identity disorder with that of gender dysphoria to emphasize that transitude is not a clinical problem in itself and that the appropriate response to “the distress that may accompany incongruence between one’s experienced or expressed gender and one’s assigned gender” is to facilitate transition.^23^ The *International Classification of Diseases 11th Revision* (ICD-11), adopted in 2019, went further by relocating its newly relabelled gender incongruence diagnosis outside of the section on mental disorders.^24^ These shifts reflect the professional consensus that transitude is a positive form of human diversity for which transition is appropriate, and that attempts to change a person’s gender identity or discourage them from transitioning is unethical.^25^ While there are risks associated with medical transition, they do not entail that being trans is a psychopathology. A wide range of risks are routinely taken on in society, including participating in sports, driving, or undergoing cosmetic surgeries — none of which are broadly conceptualized as psychopathological. The DSM-5 diagnosis of gender dysphoria does not condone conversion practices, as they are not considered appropriate responses to gender dysphoria. The body, not the mind, is the appropriate site of intervention. This view is further countenanced by materials surrounding the revision process of the DSM, which suggested preserving a diagnosis related to transitude out of a desire to ensure continued access to gender-affirming healthcare.^26^ Based on the foregoing, it cannot be suggested that trans conversion practices are a legitimate or recognized treatment for a disorder.

Proponents may concede that transitude or desiring to transition are not psychopathological but nevertheless argue that conversion practices are legitimate ways of helping consenting patients live out their lives according to their free and enlightened desires. Clinicians frequently help patients improve and bring harmony to their lives without a diagnosable condition. Under this view, conversion practices would be akin to relationship counseling or wellness programs. From the outset, this concession would be a major one. Practitioners could not rely on parental consent, could not engage in conversion practices as a condition for gender-affirming care, could not require or impose gender exploration on patients, and would have to plainly and clearly disclose the fact that they are engaging in conversion practices and that the practices are widely thought to be ineffective and harmful. It is unclear whether any conversion practices would be left.

Let us assume that some conversion practices remain. In a world devoid of transantagonism and in which conversion practices are effective in changing someone’s gender identity or desire to transition, the endeavor would perhaps warrant our respect. We do not, however, live in such a world. There is no evidence that conversion practices are effective in changing gender identity or attenuating gender dysphoria.^27^ Transantagonism and cisnormativity are pervasive in society and trans people face abhorrent rates of harassment, discrimination, and violence.^28^ Individuals are often rejected by family members when coming out as transgender and frequently internalize negative messages about transitude.^29^ Rare are those who can flourish in society unscathed. People who sought out and consented to conversion practices commonly attribute their participation to internalized prejudice or social pressures.^30^ In this social context, consent to conversion practices is suspect and healthcare providers have to consider whether the patient’s ostensible consent or putative desire reflects self-hatred or succumbing to social pressures. At a minimum, clinicians who seek to further the patient’s wellbeing and autonomy would need to rule out internalized prejudice and social pressures as motivations for pursuing conversion practices. It would not be legitimate for a clinician to offer conversion practices unless they were certain that the person’s consent reflects untainted motives, which may not be possible. Yet I would go further and question whether ostensible consent to conversion practices may ever be trusted. Unlike strife in relationships or harmony between intrinsically disparate elements of personal identity — typical concerns in counseling — there is no clear reason why one would not want to be trans other than transantagonism. If well supported and allowed access to gender-affirming care, trans people can lead normal and happy lives. Or, at least, as normal and happy as any life can be. Cisgender lives are no more intrinsically desirable than transgender ones. We have good reasons to think that all desires for trans conversion practices are rooted, directly or indirectly, in transantagonism and cisnormativity.^31^ The exercise of authority is only justified if it helps people live according to reasons that apply to them. Transantagonism and cisnormativity are no such reasons.

Proponents of conversion practices would likely challenge my suggestion that free and enlightened consent to conversion practices is impossible given the pervasiveness of transantagonism and cisnormativity. No matter, since even if conversion practices could fulfill a legitimate clinical purpose in the exceedingly implausible case where it may reflect the patient’s autonomy, they would fail to satisfy the requirements of expressive equality from the second-person viewpoint.

### Community Self-Understanding

Considering trans conversion practices from the second-person viewpoint, we must ask whether they accord with the self-understanding of trans communities. Labeling something as a mental illness, disorder, or disability and wanting to prevent it does not in and of itself constitute *prima facie* expressive inequality. Depression, for instance, is commonly understood by depressed individuals as something undesirable that they wish to be rid of. Treatments for depression are not typically seen as contrary to expressive inequality. On the contrary, they express a commitment to equality insofar as they value depressed individuals’ needs and potential for happiness. By contrast, the construction of homosexuality as a mental disorder is widely decried for reflecting prejudice and committing injustice. Whether or not trans conversion practices are *prima facie* inegalitarian from the second-person viewpoint turns on how they are understood by the people they concern — trans communities. The psychopathologization of transitude is not unethical because mental illnesses and disabilities are loathsome — lest we contribute to ableism^32^ — but because it does not cohere with trans communities’ self-understanding nor serve a legitimate clinical purpose.

The perspective of trans communities on transitude is cogently summarized by the American Psychological Association, when it explains that “diversity in gender identity and expression is part of the human experience and […] incongruence between one’s sex [assigned at birth] and gender is neither pathological nor a mental health disorder.”^33^ This is a conception of transitude that has far more in common with homosexuality than depression. Despite their heterogeneity, trans communities have long rallied against the classification of transitude as a mental disorder and vigorously opposed conversion practices as an expression of cisnormativity and transantagonism.^34^ The ICD-11 decision to move trans-related diagnoses outside of the mental health chapter occurred in response to international pressures by trans communities.^35^ To our communities, transitude is something to be proud of, something that should be honored and praised. The fact that a few understand transitude as a psychopathology to be cured by conversion practices does not negate the large number of people who understandably see these practices as an affront to their dignity.^36^ The second-person viewpoint pertains to the group targeted by a policy or practice; it is not evaluated at the individual level.

In the shared wisdom of trans communities, gender is a fundamental and stable component of personal identity that neither can nor should be changed.^37^ By contrast, attempts to alter gender identity or prevent transition in a clinical environment send the message that being who you are is somehow wrong or undesirable. This insidious message is uniquely amplified in the clinical context due to the authority of clinicians vis-à-vis disorders.^38^ As sociologist Karl Bryant has explained, trans conversion practices “made me feel that I was wrong, that something about me at my core was bad, and instilled in me a sense of shame that stayed with me for a long time afterward.”^39^

To understand how transitude may not be psychopathological despite the desire to change one’s body, consider the following analogy. Imagine that, in the cult movie Freaky Friday, cisgender teenager Anna Coleman had switched bodies with one of the cisgender boys at her school instead of switching bodies with her mother.^40^ After over a decade of identifying with her body and social classification as a girl, she is thrown into a physique that disagrees with her gendered self-image. The change would, quite reasonably, be distressing. After novelty and confusion wore off, she may even become suicidal. If she found no way of switching back bodies, she may want to socially and/or medically transition to live her life as the girl she understands herself to be. Transitioning might never bring her the sense of harmony that she had before she switched bodies, but it would doubtless help. Her hypothetical distress and desire to transition do not seem to evince a problem in her mind — on the contrary, they seem quite reasonable as responses to the situation. It is far from clear that her identification with womanhood could be undone once developed and even less clear that it should. Rather, the problem lies in her body or, more accurately, its mismatch with her gendered self-image.^41^

We can understand transitude and gender dysphoria in much the same way. The gendered self-image of trans people does not cohere with their body and/or social categorization. It matters not, for the analogy to work, what “causes” transitude and whether trans people are born this way. What matters is that once someone’s body or social categorization does not correspond to their gendered self-image, distress and discomfort are understandable reactions — even though not all trans people experience them.^42^ We need not understand distress and discomfort as problems of the mind, nor treat the desire to medically transition as evidence of such a problem. Instead, we can understand gender dysphoria and the desire to medically transition as understandable reactions to an unusual situation.

Conversion practices *prima facie* violate expressive equality insofar as they fail to conceive of transitude and transition as parts of human diversity that should not be “cured” or prevented, but respected and valued. Why change people’s gender identity or desire to transition if there is nothing wrong with it?

## Proponents Fail to Justify Conversion Practices

Appearances are defeasible. That which appears unjust may turn out not to be so upon closer examination. For proponents of trans conversion practices to overcome their *prima facie* violation of expressive equality, they would have to demonstrate that the practices’ outcomes are so much better than alternative approaches such as offering gender-affirming care that they overcome the ignominy of disparaging trans lives. The difference in outcomes would have to be sufficiently stark that even trans communities would have to concede that trans conversion practices are in their best interests despite contributing to societal transantagonism. Critically, the fact that conversion practices are *prima facie* contrary to expressive equality shifts the ethical burden of justification onto proponents of conversion practices, who must demonstrate that the practices are justified despite their inequity.

### Evaluating Outcomes

It does not suffice for trans conversion practices to have outcomes as good or no worse than other clinical approaches. If two approaches have identical outcomes in all respects except that one of them is demeaning, the demeaning one is necessarily ethically inferior. To use bioethics lingo, expressive inequality breaks clinical equipoise, making practices that may otherwise be acceptable unethical.^43^ This is reminiscent of the “minimal impairment” test in Canadian equality law, which holds that *prima facie* discriminatory laws cannot be justified if less discriminatory alternatives exist.^44^ The deeper the prima facie inequality, the more is needed to “salvage” the practice. It may be that trans conversion practices are so beyond the pale that no clinical benefits could justify them in the eyes of trans communities. For the sake of argument, I presume that this is not the case.

Again for the sake of argument, I adopt an expansive view of which sorts of evidence could demonstrate the superiority of trans conversion practices. Typical mental health outcomes such as quality of life, psychological distress, and suicidality are no doubt relevant. The little virtue ethics voice in my head — might I even say the little δαιμόνιον on my shoulder — would prefer to use εὐδαίμων or human flourishing as the ultimate standard but it is hardly one that we can measure. Physical health outcomes may be relevant to the extent they are valued by the person but we must be wary not to impose normative and idiosyncratic views of health nor value physical health over mental health. Purported evidence that conversion practices can prevent transitude is largely immaterial since it does not make for far better outcomes, though lack of such evidence would undermine its very purpose.

Outcomes attributable to transantagonism and cisnormativity are inappropriate as justifications for trans conversion practices. We do not allow reasoning of this sort in other contexts. It would be unethical to discourage homosexuality simply because it is stigmatized, and it would be morally abhorrent to the utmost than to direct parents to bleach their child’s skin to avoid racism and colorism. Allowing justifications of this kind would be tantamount to a bigot’s veto, aiding opponents of transitude in eradicating it.^45^ A bigot’s veto is all the more unacceptable given the role of trans conversion practices in legitimating and perpetuating transantagonism and cisnormativity. It could perhaps be countered that conversion practices can be justified by sufficient transantagonism — for instance in countries that systematically put trans people to death. While I do not agree, it bears pointing out that this is not the case in countries forming the majority of my readership — my writing reflects my perspective as someone who lives in Canada. It is unclear why conversion practices would be preferable to helping trans individuals live as safely as possible while affirming their gender identity, notably in private spaces. If it were conceded that extreme cases could justify conversion practices, practitioners would at a minimum be subject to a strong moral obligation to vigorously advocate for trans lives, a duty that conversion practitioners do not presently fulfill. As Leelah Alcorn tragically said upon her death by suicide after being subjected to conversion practices: “Fix society. Please.”

Data could only justify conversion practices if they are sufficiently serious, precise, and concordant to draw the inference that the practices are far superior to alternatives. Were only a subset of conversion practices shown to be beneficial, only that subset would be justified. When the data is subject to competing interpretations, interpretations that do not accord with clinical and community understandings should be preferred since conversion practices can only be justified by public reasons that respect first-person and second-person viewpoints.^46^ Idiosyncratic interpretations that reflect prejudice or arbitrariness are not enough.

### Conversion Practices are Associated with Negative Outcomes

Trans conversion practices are overwhelmingly linked to negative outcomes in the scientific literature, not positive ones. Given this evidence, it is unlikely that proponents of conversion practices could ever demonstrate that they fare far better than alternative approaches to clinical care — including immediate retirement.

In a cross-sectional study comparing trans adults who had (n = 3,869) and had not (n = 15,882) experienced conversion practices, those recalling exposure to conversion practices had 127% higher odds of lifetime suicide attempt, 59% higher odds of having attempted suicide in the last year, and 56% higher odds of having recently experienced severe psychological distress.^47^ Similar impacts on psychological wellbeing are reported in numerous other studies.^48^

Contrary to the suggestion that trans conversion practices on prepubertal children are less harmful because their gender identity is not yet stable,^49^ individuals exposed to conversion practices before the age of 10 were 315% more likely to have attempted suicide in their lifetime than those who had not experienced conversion practices.^50^ Conversion practices offered by secular professionals did not have significantly better outcomes than those offered by religious practitioners; both fared worse than the other on some measures but none of the differences reached statistical significance. Conversion practices targeting gender identity were consistently more harmful than those targeting sexual orientation, although only the difference in lifetime suicide attempts — 96% more — was statistically significant.^51^

This quantitative data is consistent with the accounts of individuals reporting exposure to trans conversion practices.^52^ Sociologist Karl Bryant stated that trans conversion practices “made me feel that I was wrong, that something about me at my core was bad, and instilled in me a sense of shame that stayed with me for a long time afterward.”^53^ His testimony resonates with the work of Robert Wallace and Hershel Russell, who explain that attempts to alter or prevent transitude or gender non-conformity can disrupt attachment and identity-formation processes, leading to shame and depression.^54^ Dr. Sé Sullivan explained that the practices “fucked me up, fueled my desires, led me to the edge of suicide more than most,” and dedicated their doctorate to “all those lost to suicide or other self-harming response to the violence” of conversion practices.^55^ Jules Sherred explained that conversion practices stopped him from transitioning for decades and led him into a series of abusive relationships because he “legitimately believed I deserved to be treated badly.”^56^ These words echo those of Erika Muse:

I feel like my life was ruined by [the conversion practitioner]. I feel like he took what could have been a great decade of life for me and turned it into a decade of depression and dysphoria. He took that from me. I mean, I feel like he destroyed me as a person.^57^

By comparison to conversion practices, countless qualitative and quantitative studies have associated gender affirmation with mental health benefits.^58^ Support for gender identity is associated with large reductions in self-harm, suicidal ideations, and suicidal attempts.^59^ Prepubescent children who are affirmed in their gender identity, which is prohibited under conversion practices, demonstrate levels of self-worth, depression, and anxiety that are comparable to cisgender peers.^60^

Healthcare guidelines and professional statements based on clinical experience and empirical evidence affirm the benefits of gender-affirming care and the harms of conversion practices with few exceptions. Access to gender-affirming care is endorsed by a wide range of leading organizations as best practices.^61^ An even greater number of organizations oppose conversion practices as harmful, ineffective, and/or unethical.^62^ Professional consensus is not conclusive proof; the scientific community has been wrong in the past. However, it provides strong evidence that conversion practices are not clearly and indubitably superior to alternative approaches — a position that no prominent organization supports at present.

Proponents of conversion practices may object, at this stage, that the available evidence is of limited quality. There are no published randomized controlled trials comparing conversion practices to other approaches. All available studies are observational and most are cross-sectional, limiting our ability to distinguish correlation from causation. Moreover, most studies recruited individuals who currently identify as transgender and thus do not account for individuals who claim to have “successfully” underwent conversion practices. If we bore the burden of justification as opponents of conversion practices, I would defend against those critiques by pointing out that the studies, while imperfect, are sufficiently diverse and convergent to establish the harms of conversion practices and comparative benefits of gender affirmation. Masked randomized controlled trials are often impossible when studying mental health outcomes and may be unethical when observational evidence is sufficiently convergent.^63^ No randomized controlled trial of parachute use has ever been conducted, yet I do not doubt that pushing someone off a plane is unethical.^64^ Causation can be reasonably inferred from the fact that respect for gender identity and expression is consistently associated with mental health benefits across a broad range of demographics, contexts, measures, and study design. While most studies did not recruit individuals from communities that report high satisfaction with conversion practices, it is noteworthy that no compelling evidence of the practices’ comparative benefits has been demonstrated in any setting.

But, as I argued, the burden of justification does not lie on opponents of trans conversion practices. The practices are *prima facie* unethical and the burden is on its proponents to justify them. Even if it could be argued that the available evidence does not prove that conversion practices are more harmful than alternatives, it is more than enough to reject the suggestion that conversion practices have far better outcomes. Given the available evidence, proponents of trans conversion practices cannot discharge their burden of justification. And since the evidence points towards harm, it is unlikely they ever could.

## Implications

My argument that proponents of trans conversion practices bear the ethical burden of justification has important implications for the law and other clinical practices. The argument may bolster medical malpractice and anti-discrimination claims as well as strengthen constitutional arguments in favor of banning conversion practices. Expressive equality may also shed light on the ethical standing of other trans health practices, gay conversion practices, surgeries on intersex newborns, applied behavioral analysis for autistic persons, and prenatal genetic counseling around disability.

### Legal Implications

The law trades in notions such as equality, dignity, proportionality, and burden of proof. These notions can be found in some shape or form throughout professional responsibility law, professional licensing law, and anti-discrimination law. Unsurprisingly given its roots in legal theory, my argument is replete with potential legal implications. These implications are relevant even in jurisdictions that expressly prohibit conversion practices since many bans only apply to minors and do not always provide a private right of action for individuals who have experienced conversion practices.^65^

My argument that proponents of trans conversion practices bear the ethical burden of justification has important implications for the law and other clinical practices. The argument may bolster medical malpractice and anti-discrimination claims as well as strengthen constitutional arguments in favor of banning conversion practices. Expressive equality may also shed light on the ethical standing of other trans health practices, gay conversion practices, surgeries on intersex newborns, applied behavioral analysis for autistic persons, and prenatal genetic counseling around disability.

Under the law of professional liability, healthcare practitioners are legally liable when their actions fall below the standard of care expected of them.^66^ The standard of care is evaluated by asking how a reasonable and competent professional would have acted in the circumstances.^67^ Insofar as a reasonable professional would act according to reason, it may be argued that they would always favor practices that do not violate the requirements of expressive inequality — which is set by reason. Faced with two alternatives that are equivalent but for the fact that one is demeaning, a reasonable professional would always choose the one that is not demeaning. This argument is strengthened in Canada, where the Supreme Court has suggested that the reasonable person cannot be ascribed prejudiced views such as transantagonism and cisnormativity.^68^ For practitioners who are not part of a regulated profession, the same argument could be made since tort law applies to professionals and non-professionals alike.

Many jurisdictions recognize an exception to the principle that professionals must respect established standards of care, known as the respectable minority rule. According to that rule, practitioners who do not meet standards of care may nevertheless be shielded from liability if they can show that they acted pursuant to a recognized and respectable minority practice.^69^ Courts often struggle to define what constitutes respectability for the purposes of the test, as it is a fundamentally normative concept rather than a popularity contest. As a New Jersey court has previously explained in relation to conversion practices, “a group of a few closely associated experts cannot incestuously validate one another as a means of establishing the reliability of their shared theories.”^70^ Expressive equality offers a compelling way of ascertaining whether practices are respectable since it is predicated on the need for public reasons, i.e. reasons that free and equal individuals might reasonably endorse. Reasons that do not reflect the requirements of expressive equality are too idiosyncratic to be respectable.

It may also be possible to shift the burden of justification onto conversion practitioners altogether, within professional liability law. Legal defenses shift the burden of justification, serving as a way for the defendant to justify or excuse a *prima facie* case. In professional liability law, courts have occasionally demonstrated a willingness to shift the burdens of justification onto clinicians — especially when proof may be uniquely difficult to adduce for the plaintiff. In *Snell v. Farrell* (1990), the Supreme Court of Canada shifted the burden of justification onto the defendant professional because the plaintiff had established a *prima facie* case of medical negligence and the professional was uniquely situated to ascertain whether her injuries had been caused by his actions.^71^ While my argument does not pertain to causation nor involve a situation where proof would be practically impossible for the plaintiff, *Snell v. Farrell* evidences an openness to rectify injustices by shifting the burden of justification. Whether courts would be willing to do the same by applying an expressive equality framework is far less certain but may be worth exploring.

Separate from professional liability law, clinicians are typically subject to codes of ethics and may be sanctioned for violating their obligations as set out under codes of ethics and licensing laws. Besides the obligation of competence, to which can be applied my argument under professional liability law, codes of ethics frequently include obligations to treat patients with dignity and respect, and not to engage in prejudicial reasoning.^72^ While many codes of ethics are not legally binding, such as those of national organizations that do not oversee licensing, they are frequently adopted by licensing bodies and are routinely applied in disciplinary proceedings. Insofar as violating the requirements of expressive equality is *ipso facto* a violation of dignity, respect for persons, and equality, my argument may be applicable in disciplinary proceedings against licensed practitioners.^73^

My argument may also bolster or form the basis of a claim under anti-discrimination law. The potential of discrimination as a cause of action for trans conversion practices has been under-explored in Canada, in part due to hesitancies in applying anti-discrimination law to regulated professionals rather than evaluating discriminatory practices under professional liability and licensing laws. However, the structure of anti-discrimination analysis resembles the structure of my argument from expressive equality. While anti-discrimination analysis varies from jurisdiction to jurisdiction, it generally follows a two-step structure. To prove discrimination, plaintiffs must first establish a *prima facie* case that the conduct or policy was discriminatory, after which defendants may try to justify their actions by showing that they adequately fulfill a legitimate purpose.^74^ Canadian law employs the language of “reasonably necessary to accomplish a legitimate purpose” whereas United States law typically asks whether the act or policy serves a sufficiently compelling societal interest.^75^ The fact that conversion practices violate the requirements of expressive equality could plausibly be interpreted as treating individuals differently on the basis of sex, gender identity, or gender expression. *Prima facie* discrimination being thence established, conversion practitioners bear the burden of justifying them. It is not enough to merely allege that the practices have not been proven harmful. They must show that engaging in conversion practices genuinely fulfills a legitimate purpose, such as preventing harm or benefitting the wellbeing of patients. Conversion practitioners cannot satisfy this requirement.

### Other Clinical Practices

The argument against trans conversion practices presented in this paper also sheds light on the ethical standing of other practices. Within trans health, it helps delineate practices that are unethical regardless of semantic debates over what counts as a conversion practice. What matters is not whether something can be properly called a conversion practice but whether it violates expressive equality. Assessment processes and eligibility criteria deployed in trans health must be carefully tailored to ensure that they do not betray double standards or reflect negatively upon transitude or transition. Even if they serve a legitimate clinical purpose, they would only be *prima facie* consistent with expressive equality if they were consistent with community self-understanding. Many prevailing practices relating to assessment and eligibility are perceived negatively by trans communities.^76^ Systematically forcing individuals to undergo so-called “gender-exploratory therapy” before accessing gender-affirming medical care would seemingly also be *prima facie* unethical because it treats transitude as suspect and/or undesirable.^77^ The approach known as “wait-and-see” or “watchful waiting” would also be *prima facie* unethical as it involves discouraging or preventing youths from socially transitioning before puberty.^78^ The same would be true of clinics that refuse to offer gender-affirming medical care to adolescents despite being qualified and competent to do so. Regardless of what we call them, the ethical standing of these practices is not dissimilar to that of conversion practices.

While my argument has centered on the experiences of trans communities, its basic structure applies to practices that pertain to other communities. The burden of justification lies on the proponents of any practice that *prima facie* does not further a legitimate clinical purpose or cohere with the concerned community’s self-understanding. In particular, conversion practices targeting queer and asexual people, non-consensual surgical procedures on intersex newborns, applied behavioral analysis for autistic individuals, and prenatal genetic counseling regarding disability appear unethical based on the argument developed in this paper.

Gay conversion practices share a history with trans conversion practices and often deployed similar interventions ranging from talk therapy to electroshocks. Though these practices target queer individuals more broadly, they remain predominantly known as “gay” conversion practices. Historically, many clinicians understood transitude as an extreme form of homosexuality, viewed homosexuality and transitude as forms of “sexual inversion,” and conceptualized them both as resulting from a disruption of normal gender development.^79^ Gender expression, rather than sexual orientation or gender identity, is often the target of conversion practices in the hopes of indirectly preventing the future development of homosexuality or transitude.^80^ Given the resemblances and intertwinement between conversion practices directed at gender identity and sexual orientation, it is clear that my argument would apply equally to the latter.

Practices seeking to change, prevent or discourage someone’s asexuality and/or aromanticism also seem to fit the bill. Asexuality refers, roughly, to the sexual orientation of individuals who experience little to no sexual attraction or desire for sexual contact, whereas aromanticism refers to the romantic orientation of individuals who experience little to no romantic attraction or desire for romantic relationships — although these definitions are contested and under constant refinement by asexual and aromantic communities.^81^ The shortened forms “ace-spec” and “aro-spec”, which stand for asexual spectrum and aromantic spectrum, include a wide range of orientations that do not involve socially normative levels of sexual or romantic attraction, including but not limited to demisexuality, graysexuality, demiromanticism, and grayromanticism.^82^ Historically, asexuality and aromanticism were predominantly seen as symptoms of hormonal or psychological disorders.^83^ Over the last few decades, however, asexual and aromantic communities have developed and disseminated understandings of asexuality and aromanticism as sexual and romantic orientations that are desirable parts of human diversity, resisting the suggestion that they should be “cured.”^84^ Asexuality and aromanticism are now commonly included in the umbrella of 2SLGBTQIA — no, the “A” does not stand for “allies.”^85^ Although the DSM-5 controversially retains diagnoses predicated on lack of sexual interest or arousal, these diagnoses can no longer be given to individuals who identify as asexual.^86^ The remaining diagnoses are in many ways comparable to the obsolete diagnosis of ego-dystonic sexual orientation, which applied to individuals who expressed distress at their sexual orientation and wished to change it, a diagnosis that was not only used to justify interventions aimed at self-acceptance but oftentimes conversion practices.^87^ No diagnosis exists for aromanticism. Given the foregoing, attempts to change, prevent, or discourage asexuality or aromanticism would appear *prima facie* contrary to the requirements of expressive equality and it is unlikely that they could be justified by evidence. Less clear is the ethical standing of practices towards consenting individuals who do not identify as asexual or aromantic and see their lack of sexual or romantic attraction as an undesirable result of trauma, hormonal imbalance, psychosocial context, or mental illness. Expressive equality is predicated on whether practices are legitimate under first-person and second-person viewpoints. From my understanding as an allosexual and alloromatic person living on the outskirts of ace-aro communities, desiring greater sexual attraction is often seen as legitimate by asexual people especially when it is a new development that clashes with the person’s longstanding personal identity.^88^ From the viewpoint of clinicians, such practices might fulfill a legitimate clinical purpose even in a world without ace-antagonism, aro-antagonism, compulsory sexuality, and amatonormativity since sexual and romantic intimacy are often central to the lives of ace and aro people’s partners, causing undesirable interpersonal tensions. However, clinicians should first help the person unlearn compulsory sexuality and amatonormativity and appreciate that intimacy and happiness do not depend on sexual or romantic desire and connection. Even then, it could be argued that attempts to change, prevent or discourage asexuality and aromanticism are always unethical on account of being inextricable from ace-antagonism, aro-antagonism, compulsory sexuality, and amatonormativity.

Non-consensual hormonal interventions and surgeries on intersex individuals, which are primarily imposed on newborns and young children, are decried as dehumanizing and harmful, paralleling the charges levied against conversion practices.^89^ While a worrisome number of clinicians continue to defend the practices, they are increasingly recognized as harmful in broader society and are opposed by many international organizations as well as by three former Surgeon Generals of the United States.^90^ These practices seek to produce sexually and gender-normative subjects regardless of the individual’s desires or self-understanding, often deploying prejudiced conceptions of gendered bodies and desirable sexual acts.^91^ These practices seem to violate the requirements of expressive equality, with evidence of alleged benefits being far from forthcoming.^92^ On the contrary, they frequently result in loss of genital sensation, chronic pain, osteoporosis, depression, and trauma, and can be experienced as a form of sexual assault by intersex youth.^93^ Clinicians should avoid assuming that intersex bodies are less desirable, respect the bodily autonomy of intersex youth, and abandon these deeply unethical practices.^94^

The most prevalent clinical approach towards autistic people is applied behavior analysis, known by the initialism ABA, which draws on behaviorist principles such as operant conditioning to modify behaviors associated with autism, notably through reinforcements and punishments. ABA reflects an assumption that autism and related behaviors are flaws that must be fixed, rather than benign differences in behavior and cognition. Philosophically, behaviorism was founded on a radical scientistic rejection of the relevance of mental content. Accordingly, ABA strives to approximate neurotypical behavior, with little regard for its impact on autistic people or their perspective. ABA targets many harmless behaviors, including a wide range of behaviors used for self-stimulation and self-regulation, which can make it even more difficult to interact with the allistic world.^95^ Reinforcements and punishments are wide-ranging, and some practitioners of applied behavioral analysis are willing to use seclusion, sensory punishments, physical punishments, and electric shocks as part of their clinical toolbox.^96^ However, ethical concerns are not limited to forms of reinforcement and punishment that are viewed as violent or extreme within the mainstream. Exposure to ABA is associated with elevated rates of post-traumatic stress disorder.^97^ Autistic communities and scholars have denounced ABA as demeaning and harmful, comparing them to conversion practices.^98^ The histories of conversion practices and ABA are intertwined through Ole Ivar Loovas, who developed ABA and coauthored core texts of the conversion practices literature.^99^ His approach to autistic youth, however, was “markedly more brutal” than his approach to gender non-conforming youth.^100^ While the clinical community continues to view ABA as furthering a legitimate clinical purpose, this assumption seems to reflect prejudice towards autism and does not cohere with the self-understanding of autistic communities, who see autism as a desirable form of human diversity.^101^ Autistic scholars and advocates have notably argued that autistic people’s “tendency to direct communication and disinterest in social norms reduces the likelihood that cisgenderism and transphobia will prevent them from disclosing their identities,” situating autism as offering partial protection against prejudice and oppressive socialization. Though autistic people face high levels of discrimination and are not immune to social norms nor the punishments for violating said norms, the argument highlights some of the positive facets of autism.^102^ Society would benefit if neurotypical people were more straightforward and less bound by social norms. The conceptual and empirical foundations of clinicians’ attachment to ABA are suspect.^103^ In any case, the fact that ABA conflicts with autistic communities’ self-understanding suffices to *prima facie* violate the requirements of expressive equality. The fact that many ABA practitioners allegedly no longer use aversive techniques is immaterial, as it is *prima facie* unethical to discourage or “extinguish” value-neutral autistic communication — such as flapping and echolalia.^104^ Autistic communities express a desire for more research and interventions that focus on overcoming discrimination and addressing deficits in practical skills on their own terms, including with regards to making friends, finding employment, navigating transit, and finding accessible healthcare. None of that requires ABA. Since ABA runs afoul of expressive equality, the burden of justification is borne by its proponents. Given the available evidence, it is doubtful that they could discharge it.

Lastly, my argument may have implications for prenatal genetic counseling. Prenatal genetic counseling has been criticized as an ableist and eugenic practice by disabled communities and scholars, as it is often used to prevent or select against the birth of disabled children.^105^ Prospective parents and clinicians commonly equate disability with suffering and unhappiness, casting them as lives less worth living in spite of contrary evidence.^106^ These assumptions reflect a wilful ignorance of disabled people’s perspectives, betraying ableist biases. While preventing certain specifc conditions may be generally understood as desirable by those who have them, many disabled communities understand their impairments as desirable forms of human diversity and are opposed to attempts to “cure” or prevent them.^107^ This self-understanding is manifest in the social model of disability, which conceptualizes disability not as inherent to impairment but instead as partly or completely a result of the social environment. Being Deaf, for example, would not be a disability in a society where sign language is the primary means of communication. While certain conditions like chronic pain or depression are something that people may wish to be rid of and could be legitimate targets of prenatal genetic counseling, prenatal genetic counseling is indiscriminately applied to a wide range of disabilities that disabled communities do not see as inherently negative and may take pride in. Preventing those disabilities before the person is even born — voiding any possibility of consent and autonomy — reflects a negative moral assessment that *prima facie* violates the requirements of expressive equality from the second-person standpoint.^108^ More often than not, prenatal genetic counseling reflects prospective parents’ desire for comfort rather than a genuine concern for the hypothetical child’s perspective and subjective wellbeing.^109^ Even when prospective parents engage in prenatal genetic counselling out of concern for the child, they often incorporate prejudices and values that do not reflect the understanding of disabled communities.^110^ For instance, children with Down syndrome demonstrate much lower levels of anxiety and depression than the general population and report comparable levels of positive and negative emotions.^111^ In spite of this evidence, people continue to view Down syndrome as inherently negative. The right to abortion regardless of motive is morally robust and, I would argue, absolute. The same cannot be said of prenatal genetic counselling.

## Conclusion

Transgender conversion practices neither fulfill a legitimate clinical purpose nor respect trans communities’ understanding of transitude as a desirable form of human diversity. Given their *prima facie* injustice, the burden of justification for conversion practices shifts — nay, is *trans*ported — onto their proponents. To salvage the practices, proponents would have to show that they are far more beneficial than alternative clinical approaches. The evidence of benefits must be compelling enough to outweigh the *prima facie* violation of expressive equality in the eyes of trans communities. That burden cannot be discharged on the available evidence. On the contrary, the evidence suggests if not altogether proves that trans conversion practices are harmful, shedding considerable doubt as to whether future evidence could plausibly justify them. The argument presented in this paper could bolster legal claims against conversion practitioners under professional liability law, professional licensing law, and anti-discrimination law.^112^ It also applies, *mutatis mutandis*, to practices targeting sexual orientation, asexuality and aromanticism, intersex traits, autism, and disabilities, shedding doubts on the ethicality of various conversion-like practices. It is not on marginalized communities to show that stigmatizing practices are unethical. It is on the practices’ proponents to justify them. And they cannot.

## Note

The author has no conflicts to disclose.
